# Influence of sex on the outcomes of uniportal video‑assisted thoracoscopic sympathicotomy for primary palmar hyperhidrosis

**DOI:** 10.20452/wiitm.2025.17934

**Published:** 2025-02-10

**Authors:** Turkan Dubus, Gokce Cangel, Fatih Kesmezacar

**Affiliations:** Department of Thoracic Surgery, Health of Science University, Basakşehir Cam and Sakura City Hospital, Istanbul, Turkey; Department of Thoracic Surgery, Haseki Training and Research Hospital, Istanbul, Turkey; Imaging Program, Vocational School of Health Services, Istanbul University‑Cerrahpasa, Istanbul, Turkey

**Keywords:** palmar hyperhidrosis, sex differences, T3 and T3–T4 sympathicotomy, uniportal video‑assisted thoracoscopic sympathicotomy

## Abstract

**INTRODUCTION:**

Primary palmar hyperhidrosis (PPH) impairs the quality of life. Video‑assisted thoracoscopic sympathicotomy is an effective treatment method; however, the impact of sex on surgical outcomes accord‑ ing to the denervation level (T3 vs T3–T4 sympathicotomy) remains unclear.

AIM This study investigated the efficacy, complications, and symptom relief rates of isolated T3 vs combined T3–T4 sympathicotomy for PPH, focusing on sex differences.

**MATERIALS AND METHODS:**

A retrospective analysis of 327 patients undergoing bilateral uniportal video‑assisted thoracoscopic sympathicotomy for PPH between 2012 and 2022 was performed. The patients were divided into 2 groups depending on the procedure type (isolated T3 sympathicotomy [n = 167] vs T3–T4 combined sympathicotomy [n = 160]). Demographic data, procedure outcomes, and complications were compared.

**RESULTS:**

Success rates were 95.8% in the T3 sympathicotomy group and 93.8% in the T3–T4 sympathicotomy group, with no significant difference. The most common complication was dryness of the hands. The overall complication rate was lower in the T3 than in the T3–T4 sympathicotomy group (9.6% vs 14.4%; P = 0.04). Compensatory sweating occurred in 2.4% and 3.1% of the participants in the T3 and T3–T4 sympathicotomy groups, respectively (P = 0.52). The frequency of compensatory sweating, chest pain, and dryness of the hands was significantly higher in men. Age, sex, and duration of surgery had no independent influence on the occurrence of complications.

**CONCLUSIONS:**

Isolated T3 sympathicotomy is an effective and safe option for the treatment of PPH, and is associated with fewer complications than combined T3–T4 sympathicotomy. Higher complication rates in men emphasize the need for sex‑specific surgical planning and patient counseling.

## INTRODUCTION 

Primary palmar hyperhidrosis (PPH) is a chronic disorder characterized by excessive sweating of the palms, typically presenting in adolescence or early adult‑ hood. Individuals affected by this condition face considerable challenges in their social and professional lives. Persistence of symptoms may lead to a loss of self‑confidence, social isolation, and occupational limitations, all of which have a significant impact on the quality of life. The prevalence of PPH in the general population is estimated at 1%–3%, with higher rates observed in younger and more physically active groups.^1^

Although the exact cause of PPH is not fully understood, it has been associated with hyperactivity of the sympathetic nervous sys‑ tem. Treatment options can be divided into nonsurgical and surgical. Nonsurgical treatments include topical antiperspirants, botulinum toxin injections, and iontophoresis. While these methods provide temporary relief, especially in mild‑to‑moderate cases, they are of‑ ten not a long‑term solution, as they require repeated use.^2^

On the other hand, surgical treatments offer permanent solutions for PPH. Procedures such as sympathicotomy and sympathectomy target the sympathetic chain, and are known for their long‑term effectiveness. During sympathectomy, a portion of the nerve chain is permanently removed, while sympathicotomy involves severing the chain without its removal. In recent years, uniportal video‑assisted thoracoscopic surgery (U‑VATS) has become increasingly accepted as a minimally invasive surgical method for sympathicotomy. However, there are limited literature data on the optimal lev‑ el of sympathetic chain interruption (isolated T3 sympathicotomy or combined T3–T4 sym‑ pathicotomy) and the influence of patient sex on surgical outcomes.^3^^,^^4^

**FIGURE 1:**
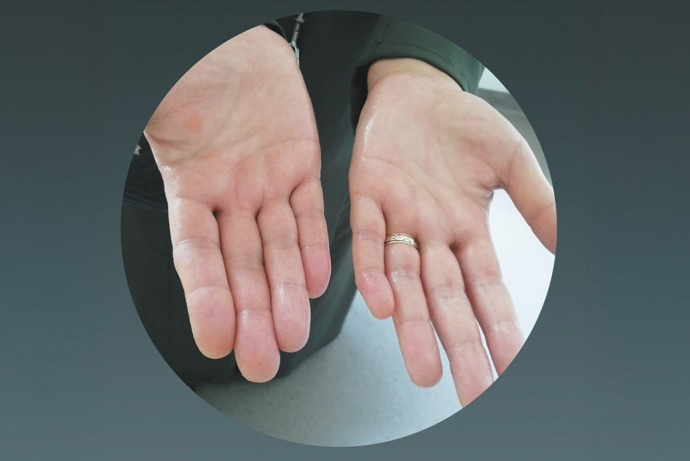
Image showing palmar hyperhidrosis

**FIGURE 2:**
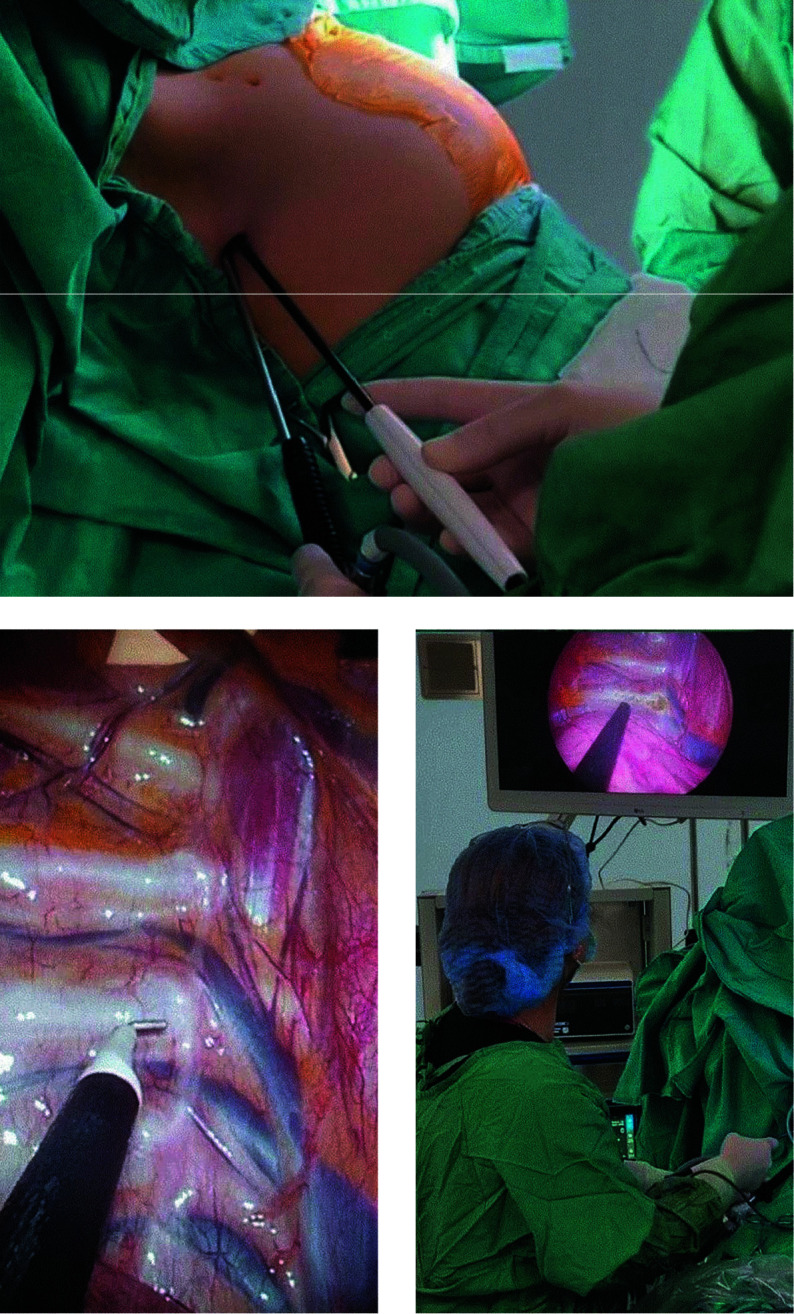
Images showing uniportal video‑assisted thoracoscopic sympathicotomy (intraoperative view and positioning of the instruments)

Existing studies have generally compared the ef‑ ficacy and complications of isolated T3 and combined T3–T4 sympathicotomy; however, the impact of sex differences on surgical outcomes has not been adequately investigated. This gap represents a significant limitation in surgical planning and patient counseling.^5^

## AIM

The aim of this study was to evaluate and compare the efficacy, complication rates, and prevalence of symptom relief of isolated T3 and combined T3–T4 sympathicotomy. In addition, the impact of sex on surgical outcomes was investigated. By providing evidence‑based insights into the comparative safety and efficacy of these procedures, this study aimed to help clinicians select the optimal surgical approach, particularly taking into account sex‑specific risk factors and long‑term outcomes.

## MATERIALS AND METHODS

Patients This was a retrospective analysis of 327 patients with PPH who underwent a single‑session bilateral U‑VATS sympathicotomy at our hospital between November 2012 and May 2022. The patients were divided into 2 groups: those undergoing isolated T3 sympathicotomy (n = 167; group 1) and those under‑ going combined T3–T4 sympathicotomy (n = 160; group 2). We collected data on patient demograph‑ ics as well as the rates of treatment success, com‑ plete symptom resolution, recurrence, and com‑ plications. The data were extracted from hospital records, surgical reports, outpatient follow‑up re‑ ports, and discharge notes.

Patients aged 18 to 50 years, with a clinical diagnosis of PPH confirmed by medical history and physical examination were included FIGURE 1. Exclusion criteria comprised previous thoracic surgery, significant comorbidities (eg, severe cardiovascular or pulmonary disease), and secondary hyperhidrosis due to a systemic or metabolic disease.

### Surgical procedure

All patients were placed in the semi‑Fowler position, with arms bent at 90 degrees, and intubated with a double‑lumen endobronchial tube. Sympathicotomy was performed bilaterally during the same session through a 5‑mm incision in the third intercostal space, along the midaxillary line. It involved careful dis‑ section and transection of the sympathetic chain, and electrocautery ablation of the Kuntz nerves. There were no technical differences between the T3 and T3–T4 procedures FIGURE 2.

**TABLE 1:** Patient demographic data and treatment outcomes

Parameter	T3 sympathicotomy	T3–T4 sympathicotomy	*P* value
	(n = 167)	(n = 160)	
Age, y, mean (SD)	28.6 (6.2)	29.1 (6)	0.67
Sex Men	98 (58.7)	94 (58.8)	0.82
Women	69 (41.3)	66 (41.2)	
Duration of surgery, min, mean (SD)	15 (4)	20 (5)	0.05
Duration of hospitalization, d	1 (1–1)	1 (1–1)	0.08
Treatment success	160 (95.8)	150 (93.8)	0.23
Complete relief of symptoms	155 (92.8)	146 (91.3)	0.18
Symptom recurrence	5 (3.0)	7 (4.4)	0.31
Complications	16 (9.6)	23 (14.4)	0.04

Data are presented as number (percentage) or median (interquartile range) unless otherwise indicated.

P values <0.05 were considered significant.

Postoperatively, the intrathoracic air was aspirated with a 12‑F chest catheter. There were no intraoperative complications, and all patients recovered quickly without the need for intensive medical care. No patient required placement of a chest drain or prolonged hospitalization due to serious postoperative complications.

### Statistical analysis 

Categorical variables were presented as frequencies and percentages, while continuous variables were expressed as means with SD (for normally distributed data) or medians with interquartile ranges (for non‑normally distributed data). The Levene test was used to assess homogeneity between the groups. The χ2 test was used to analyze categorical data, including a com‑ parison of the overall complication rates between the groups, while the t test or Mann–Whitney test was used to compare continuous variables, de‑ pending on the normality of distribution. Logistic regression analysis was performed to assess the in‑ dependent effects of age, sex, and duration of sur‑ gery on the complication rates. Odds ratios (ORs) with 95% CIs were reported for each predictor.

The effect size was measured using the Cohen d, which classifies the effect size as small (<0.2), me‑ dium (~0.5), or large (>0.8). Significance was de‑ fined as a P value below 0.05. All analyses were performed using the SPSS software, version 25.0 (IBM Corp., Armonk, New York, United States).

### Ethical approval

This study was conducted in accordance with the Declaration of Helsinki and approved by the Ethics Committee of the Istanbul Training and Research Hospital (1678). Informed consent was obtained from all patients prior to their inclusion in the study. Patient confidentiality was strictly maintained throughout the study and all identifying information was anonymized during data analysis.

## RESULTS

In this retrospective study, we analyzed 167 patients who underwent isolated T3 sympathicotomy (group 1) and 160 patients who underwent combined T3–T4 sympathicotomy (group 2). Demographic data, treatment success rates, rates of complete symptom resolution, as well as recurrence and complication rates were compared between the groups.

The group 1 comprised 98 men (58.7%) and 69 women (41.3%) at a mean (SD) age of 28.6 (6.2) years. In the group 2, there were 94 men (58.8%) and 66 women (41.2%), and the mean (SD) age was 29.1 (6) years. No differences were found between the groups in terms of age or sex distribution TABLE 1.

The mean (SD) duration of the procedure was 20 (5) minutes in the group 1 and 22 (6) minutes in the group 2 (Cohen d = 0.35; P = 0.05). The duration of hospitalization was similar in both groups, with a maximum length of stay of 1 day (P = 0.08). The treatment success rate was 95.8%

in the group 1 and 93.8% in the group 2 (Cohen d = 0.13; P = 0.23). Complete resolution of symptoms was achieved in 92.8% of the patients in the group 1 and 91.3% in the group 2 (Cohen d = 0.15; P = 0.18). These results show that both approaches are highly effective but isolated T3 sympathicotomy has slightly higher success and symptom resolution rates.

The postoperative recurrence rates were similar in the groups (3% in the group 1 vs 4.4% in the group 2; Cohen d = 0.11; P = 0.31), suggesting that the methods are comparable with respect to long‑term results.

The observed complications included compensatory sweating, pneumothorax, hemothorax, skin infections, chest pain, dryness of the hands, and numbness in the fingers. The most common com‑ plication in both groups was hand dryness, with a prevalence of 4.5% in the group 1 and 6% in the group 2 (Cohen d = 0.08; P = 0.02). Compensatory sweating was observed in 2.5% of the patients in the group 1 and 3% of the patients in the group 2 (Cohen d = 0.09; P = 0.52).

**FIGURE 3:**
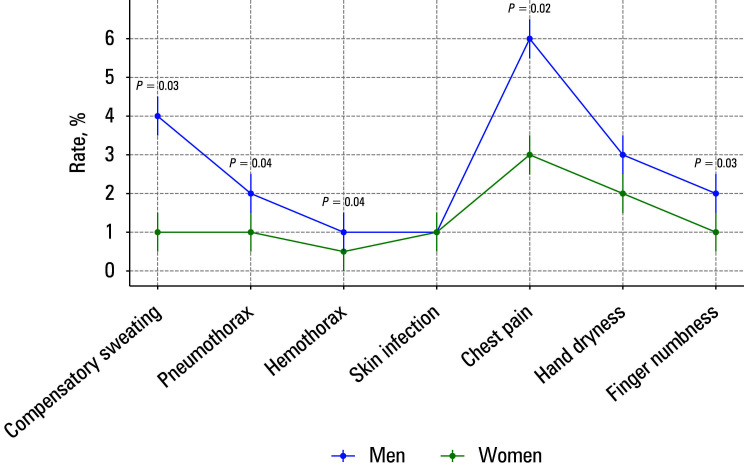
Distribution of complication following T3 and T3–T4 sympathicotomy procedures by sex. The error bars indicate the standard error for each rate.

The overall complication rate was lower in the group 1 than in the group 2 (9.85% vs 14.1%; Cohen d = 0.35; P = 0.04), suggesting that isolated T3 sympathicotomy is clinically safer TABLE 2. The overall complication rate was significantly higher in men. In particular, compensatory sweat‑ ing (Cohen d = 0.4), chest pain (Cohen d = 0.35), and hand dryness (Cohen d = 0.38) were more frequent and more severe in the male cohort FIGURE 3. This can be attributed to a higher activity of the sympathetic nervous system in men.

Multivariable logistic regression analysis examined the combined effects of age, sex, and duration of surgery on the likelihood of complication occurrence. None of these variables was identified as a significant predictor of complications. The ORs were 1.004 (95% CI, 0.951–1.061) for age, 0.986 (95% CI, 0.504–1.931) for sex, and 0.996 (95% CI, 0.936–1.061) for duration of surgery TABLE 3.

**TABLE 2:** Distribution of complications

Complication	T3 sympathicotomy	T3–T4 sympathicotomy	*P* value	Men	Women	*P* value
	(n = 167)	(n = 160)		(n = 192)	(n = 135)	
Compensatory sweating	4 (2.4)	5 (3.1)	0.52	7 (3.65)	2 (1.48)	0.03
Pneumothorax	1 (0.6)	2 (1.25)	0.6	2. (1.04)	1 (0.74)	0.04
Hemothorax	1 (0.6)	1 (0.63)	0.85	1 (0.52)	1 (0.74)	0.04
Skin infection	1 (0.6)	2 (1.25)	0.5	1 (0.52)	1 (0.74)	0.05
Chest pain	3 (1.8)	4 (2.5)	0.6	6 (3.12)	2 (1.48)	0.03
Hand dryness	7 (4.2)	10 (6.25)	0.04	12 (6.25)	5 (3.7)	0.02
Finger numbness	2 (1.2)	2 (1.25)	0.68	4 (2.8)	2 (1.48)	0.03

Data are presented as number (percentage) of patients.

P values <0.05 were considered significant.

**TABLE 3:** Multivariable logistic regression analysis of factors associated with the occurrence of complications

Parameter	β coefficient	OR	95% CI	*P* value
Age	0.004	1.004	0.951–1.061	0.88
Sex	–0.014	0.986	0.504–1.931	0.97
Duration of surgery	–0.004	0.996	0.936–1.061	0.91

Abbreviations: OR, odds ratio

## DISCUSSION

Primary hyperhidrosis affects about 0.6%–1% of the population and is often characterized by excessive sweating of the hands. Thoracic sympathectomy was first introduced in the 1930s, with Kux presenting significant advances in this technique in 1954.5 Since then, sympathectomy and sympathicotomy have become standard treat‑ ment methods for this condition. With the introduction of VATS in the 1990s, thoracic surgery evolved toward minimally invasive techniques.6 Among these, sympathicotomy is a faster and equally effective alternative to sympathectomy, with a similar safety profile.^7^^,^^8^

Despite its effectiveness, compensatory sweating remains a common and problematic side effect of sympathicotomy, with a reported incidence of 3%–98%, depending on the patient group and surgical approach.2 Other complications, such as Horner syndrome, recurrence, bleeding, and pneumothorax, are less common and occur in around 3%–10% of cases.^9^ The average duration of sur‑ gery reported in the literature is 30–60 minutes, whereas in our study, it was significantly shorter, at 20 minutes (range, 15–30 minutes).^10^,^11^ This difference could be due to procedural refinements and the use of a single‑port technique, which has been shown to reduce the complexity of the procedure and recovery time.

Thoracic sympathectomy is widely regarded as a safe and effective treatment for PPH, providing relief in 90%–100% of patients. However, relapse occurs in up to 10% of cases, possibly due to anatomical variants, such as the Kuntz nerve or rami communicantes. Treating these branches near the T2, T3, and T4 ganglia can reduce the recurrence rate. However, procedures involving the T2 ganglion carry a significantly higher risk of compensatory sweating and, therefore, are less beneficial.^12^ The Society of Thoracic Surgeons recommends T3 or T4 sympathectomy for PPH, and points out that procedures performed at the T4 level carry a lower risk of compensatory sweating, but can lead to increased sweating of the palm. T4 or T5 incisions are often recommended for patients with combined hand and foot sweating.^13^

Our study is consistent with certain aspects of the previous research, but also points to contextual differences. Zhang et al^14^ reported that isolated T4 sympathicotomy reduced the rates of compensatory sweating, hand dryness, and gustatory sweating, relative to isolated T3 procedures. In contrast, our results showed that isolated T3 sympathicotomy had a lower overall complication rate than combined T3–T4 sympathicotomy without compromising efficacy. Xie et al^15^ observed lower rates of compensatory sweating after isolated T4 sympathicotomy, whereas our analysis of combined T3–T4 procedures revealed higher complication rates, especially for compensatory sweating and dryness of the hands. These apparent discrepancies may be due to differences in surgical focus (isolated vs combined procedures), patient demographics, or surgical techniques. For example, Zhang et al^14^ compared only isolated T3 and T4 procedures, whereas our study also focused on combined T3–T4 procedures, which may be associated with greater risks. This distinction emphasizes the importance of tailoring the surgical approach to the specific patient profile and anatomic conditions.

Yang et al^16^ found that combined T3–T4 sympathicotomy for plantar hyperhidrosis achieved higher success rates but, at the same time, resulted in higher complication rates, as compared with isolated T3 sympathicotomy. Our study did not fo‑ cus on plantar hyperhidrosis; however, we found that isolated T3 sympathicotomy was sufficient to achieve symptom relief in palmar hyperhidro‑ sis cases, highlighting the importance of minimizing unnecessary surgical risks. Huang et al^17^ also reported a reduction in compensatory sweating and dryness of the hands with T4 procedures, but noted increased palm moisture—a finding con‑ sistent with our observations of higher complication rates for the procedures involving resection at the T4 level.

A major strength of our study is the identification of sex‑specific differences in surgical outcomes. We noted significantly higher rates of com‑ plications among men, including compensatory sweating, chest pain, and hand dryness. This observation supports the hypothesis that men may have increased sympathetic nervous system activity, as proposed in previous studies.^18^ In addition, Sang et al^19^ suggested that avoidance of T2 sympathicotomy may reduce the risk of compensatory sweating and Horner syndrome, which is consistent with our findings that isolated T3 sympathicotomy has a more favorable safety profile. Similarly, Dogru et al^20^ found that while T2–T4 sympathicotomies can improve quality of life, isolated T3 sympathicotomy results in fewer complications. These findings underscore the importance of individualizing the surgical approach, incorporating sex‑specific considerations, and focusing on choosing optimal levels of intervention to improve patient‑related outcomes. They highlight the significant influence of sex on the risk of complications, which should be carefully considered in clinical decision‑making. This study emphasizes the important role of sex in determining postoperative outcomes of U‑VATS sympathicotomy for PPH. We found that postoperative complications, particularly compensatory sweating, chest pain, and dryness of the hands, are more common in men. These differences may be attributed to sex‑specific differences in autonomic nervous system activity. Previous studies have reported that men tend to show a stronger sympathetic nervous system response than women, which could lead to more pronounced postoperative side effects. In addition, differences in skin structure, sweat gland distribution, and hormon‑ al balance may contribute to the observed variability in surgical outcomes.^21^^,^^22^^,^^23^

It has been suggested that women are generally more satisfied with the outcomes of sympathicotomy, possibly due to a lower prevalence of severe compensatory sweating in female patient populations. Some researchers have hypothesized that estrogen‑induced modulation of the sympathetic chain activity may have a protective effect against excessive postoperative reactions. In addition, anatomical differences, including variations in nerve branching patterns, could influence the effectiveness of the surgical procedure between the sexes.^23^

Tailored approaches, such as changes in the extent of sympathicotomy or the use of alternative techniques (eg, stapled sympathectomy), could help reduce the risk of complications in men.^24^ Future studies with larger sample sizes and longer follow‑up periods are needed to further elucidate the role of sex in determining the long‑term success and safety of sympathicotomy procedures. Shorter operative time associated with T3 sympathicotomy not only increases patient safety, but also improves the efficiency of the procedure, making it a practical choice for high‑volume centers and outpatient facilities. Given the higher predisposition to postoperative complications in men, tailored preoperative counseling and risk mitigation strategies are recommended for this patient group.

Furthermore, our results suggest that performing sympathectomy at the T4 level for palmar hyperhidrosis does not provide significant benefits, but increases the risk of complications. For patients with concomitant hand and foot hyperhidrosis, further investigation is needed to deter‑ mine the optimal level of intervention that would minimize the risk of complications.

Although our study provides clear evidence of the safety and efficacy of T3 sympathicotomy, further research is warranted in several areas. Higher complication rates in men emphasize the need for studies to investigate the physiological or an‑ atomical factors underlying these differences. In addition, long‑term follow‑up studies are needed to investigate recurrence rates, patient satisfac‑ tion, and improvement in quality of life. The inclusion of patient‑reported outcomes in future studies could provide a more holistic perspective on successful treatment.

In addition, development of advanced surgical techniques or technological innovations, such as robotic thoracoscopy, could improve the precision and outcomes of these procedures. Research into preoperative assessments or imaging techniques that would help predict complications and optimize surgical planning could also prove valuable. This study contributes to the growing body of knowledge on the optimal surgical treatment of PPH. In contrast to previous studies that emphasized the potential benefits of T4 sympathicotomy or combined T3–T4 procedures, our results show that similar or better results with fewer complications can be achieved with isolated T3 sympathicotomy. This study also highlights the im‑ portance of sex‑specific considerations in surgical planning, filling a gap in the current literature.

## CONCLUSIONS

Isolated T3 sympathicotomy is an effective and safe treatment option for PPH, with high success rates and fewer complications, as compared with combined T3–T4 procedures. Addition of T4 ganglion resection increas‑ es the risk of complications, such as dryness of the hands and compensatory sweating, with no clear efficacy benefit.

Higher complication rates in men emphasize the importance of considering sex‑specific factors in surgical planning. Isolated T3 sympathicotomy should be the preferred approach for most patients, while further research is needed to investigate sex differences and long‑term outcomes.
